# Essential Oils as an Antifungal Alternative for the Control of Various Species of Fungi Isolated from *Musa paradisiaca*: Part I

**DOI:** 10.3390/microorganisms13081827

**Published:** 2025-08-05

**Authors:** Maritza D. Ruiz Medina, Jenny Ruales

**Affiliations:** Departamento de Ciencias de Alimentos y Biotecnología (DECAB), Escuela Politécnica Nacional (EPN), Quito 170143, Ecuador; jenny.ruales@epn.edu.ec

**Keywords:** *Origanum vulgare*, *Salvia rosmarinus*, *Syzygium aromaticum*, *Thymus vulgaris*, *Ocimum basilicum*, *Cinnamomum verum*, antifungal alternative, post-harvest management, banana

## Abstract

This study evaluated the antifungal potential of essential oils (EOs): oregano (*Origanum vulgare*), rosemary (*Salvia rosmarinus*), clove (*Syzygium aromaticum*), thyme (*Thymus vulgaris*), cinnamon (*Cinnamomum verum*), and basil (*Ocimum basilicum*). These oils were tested against fungi isolated from banana peels (*Musa paradisiaca*). The fungi tested were identified through macroscopic and microscopic analyses and DNA sequencing, after being isolated in potato dextrose agar (PDA) medium modified with 0.05% chloramphenicol. Subsequently, the antifungal properties of the tested essential oils were evaluated *in vitro* at concentrations of 200, 400, 600, 800, and 1000 ppm prepared in a 0.05% Tween 80 solution. Cinnamon EOs showed the highest antifungal activity, significantly inhibiting the growth of pathogens at a concentration of 400 ppm. Other EOs showed moderate effects at higher concentrations: rosemary inhibited fungal growth at 600 ppm, oregano at 800 ppm, and clove at 1000 ppm. These findings highlight the potential of EOs as eco-friendly alternatives to synthetic fungicides, contributing to the development of sustainable agricultural practices and the post-harvest management of bananas. It is recommended to conduct future research to assess the economic viability and practical impacts of large-scale applications.

## 1. Introduction

Bananas (*Musa paradisiaca*) are exotic and climacteric fruits believed to have originated in Southeast Asia, particularly in regions such as India, Malaysia, Indonesia, and the Philippines. Depending on the region, they are commonly known as banana or guineo [1,2]. In Ecuador, the province of El Oro, Machala, has been the top exporter since 1910 to the United States, Peru, and Chile, standing out for its internationally recognized quality [3]. The fruit is incomparable to others offered at lower prices from other countries [4,5]. It is consumed when the peel is yellow and is an important source of vitamins, potassium, carbohydrates, and minerals [6].

Bananas are one of the most important crops worldwide due to their economic and nutritional value. As a climacteric fruit, it is highly perishable, and post-harvest losses are primarily attributed to fungal infections [7]. Among the most important pathogens are *Colletotrichum* spp., *Lasiodiplodia* spp., *Fusarium* spp., and *Aspergillus* spp., which cause diseases such as anthracnose, crown rot, and root rot caused mainly by *Colletotrichum* spp. [8,9], resulting in substantial economic losses for producers. Despite advances in post-harvest management, synthetic fungicides remain the primary method for controlling these pathogens, raising concerns about environmental impact, fungicide resistance, and consumer health [1,2].

In recent years, there has been a growing interest in the use of plant-derived compounds, such as EOs, as natural antifungal agents. EOs from plants such as oregano (*Origanum vulgare*), cinnamon (*Cinnamomum verum*), and clove (*Syzygium aromaticum*) are rich in bioactive compounds such as carvacrol, cinnamaldehyde, and eugenol, which have demonstrated antimicrobial properties [10,11]. However, their specific efficacy against fungal pathogens affecting bananas remains unexplored, particularly under *in vitro* and *ex vivo* conditions.

This study aims to evaluate the antifungal efficacy of commercially available EOs against pathogenic fungi isolated from banana peels. The research contributes to the development of sustainable alternatives to synthetic fungicides, supporting ecological agricultural practices and improving post-harvest management. The composition of the EOs used in the study is to understand the bioactive compounds responsible for the observed antifungal activity.

Oregano EO (*Origanum vulgare*) stands out for its high carvacrol content (60–80%), a phenolic compound with potent antimicrobial and antioxidant properties. Other important components include thymol (5–10%), p-cimeno (5–10%), and γ-terpinene (2–5%). These compounds act synergistically to inhibit the growth of microorganisms [12]. Rosemary EO (*Salvia rosmarinus*) contains mainly 1,8-cineole (20–50%), carnosol (5–15%), rosmarinic acid (2–5%), and α-pinene (10–20%), all known for their antioxidant, antimicrobial, and potential neuroprotective effects [13].

Clove EO (*Syzygium aromaticum*) is characterized by its high concentration of eugenol (70–85%), followed by acetyl eugenol (5–15%) and β-caryophyllene (5–12%). This chemical profile makes it an effective agent against fungi and bacteria [14]. Likewise, thyme EO (*Thymus vulgaris*) has thymol (30–50%) and carvacrol (10–20%) as its predominant compounds, complemented with p-cymene (15–25%) and γ-terpinenes (10–15%) [15]. These compounds are responsible for its antifungal capacity and use in biological applications.

Cinnamon EO (*Cinnamomum verum*) has cinnamaldehyde (60–70%) and eugenol (5–15%) as its main components, accompanied by trace amounts of linalool (3–5%). These compounds provide a wide range of antimicrobial and antioxidant properties [14]. Finally, basil EO (*Ocimum basilicum*) is distinguished by its high eugenol content (50–70%), linalool (10−15%), and methyl chavicol (5–15%), compounds with antimicrobial, anti-inflammatory, and antioxidant properties [16].

The antifungal effects of EOs are mainly explained by their ability to disrupt fungal cell membranes and by their interference with essential metabolic processes. For example, compounds like carvacrol and thymol, present in oregano (*Origanum vulgare*) and thyme (*Thymus vulgaris*), are integrated into the lipid bilayer of fungal cell membranes, increasing their permeability. This causes the loss of ions, metabolites, and essential proteins, ultimately leading to cell death [12,17].

Cinnamaldehyde and eugenol, present in cinnamon EOs (*Cinnamomum verum*) and clove (*Syzygium aromaticum*), act by denaturing intracellular proteins, including enzymes essential for cellular metabolism, which interferes with fundamental processes such as cellular respiration and ATP synthesis [11,18]. Additionally, some compounds like rosmarinic acid, present in rosemary (*Salvia rosmarinus*), and eugenol, present in basil (*Ocimum basilicum*), induce the generation of reactive oxygen species (ROS). These ROS cause oxidative damage to DNA, proteins, and lipids of cellular membranes, accelerating programmed cell death [13,19,20].

Another significant mechanism is the inhibition of ergosterol biosynthesis, a critical component of the fungal cell membrane. Cinnamaldehyde and carvacrol directly disrupt this pathway, compromising the membrane structure and impairing nutrient transport [10,15]. The phytotoxicity of EOs can be influenced by their chemical composition and the concentration used. Compounds such as eugenol, thymol, and carvacrol, although effective as antimicrobials, can cause damage to plant tissues at high concentrations [21]. Previous studies have shown that the direct application of EOs or concentrated emulsions can affect the physiology of plants, causing chlorosis, necrosis, or reduced tissue growth [10,22]. Therefore, it is essential to determine the optimal concentration that maximizes antifungal efficacy without compromising the integrity of plant tissues.

Some EOs, such as cinnamon oil (*Cinnamomum verum*) and clove (*Syzygium aromaticum*), have shown low phytotoxicity in specific applications when used in controlled concentrations. It was reported that emulsions of 0.1–0.5% can be safe for applications on post-harvest fruits and vegetables [11], though the sensitivity of crops can vary. This highlights the need for specific testing on the agricultural products intended for protection.

The genus *Cladosporium* spp. belongs to the phylum *Ascomycota* and the class *Dothideomycetes*. It is widely known for its presence in both indoor and outdoor environments. Microscopically, it is characterized by septate hyphae and conidia arranged in chains or clusters. The conidia are generally black, olive green, or brown, with globular or elliptical shapes, and the hyphae exhibit dark branching, a rough texture, and a cottony or velvety appearance. This genus includes saprophytic and pathogenic species, commonly involved in the colonization of plant tissues and the induction of diseases such as leaf spots and fruit rots. Additionally, some species of *Cladosporium* are potential allergens and may cause respiratory problems in humans, particularly in sensitive individuals [23,24].

The genus *Lasiodiplodia* spp. belongs to the phylum *Ascomycota* and class *Dothideomycetes* and is widely associated with diseases in fruit crops, particularly fruit rot. Microscopically, this genus is characterized by conidia that are elliptical or spherical in shape, which may occasionally form chains. The hyphae are septate, branched, and often bear conidia attached to their spores. The texture of the colonies is usually cottony or velvety, with colors ranging from dark brown to black. These morphological characteristics allow for precise identification through microscopic analysis. *Lasiodiplodia* spp. mainly impacts agriculture, causing significant economic losses by affecting the quality and shelf life of fruits [25,26].

Anthracnose is a common disease in many plants, caused by pathogenic fungi of the genus *Colletotrichum* [27]. This genus belongs to the phylum *Ascomycota* and class *Sordariomycetes* and is recognized as one of the main agents of diseases in agricultural crops. Microscopically, *Colletotrichum* spp. are characterized by cylindrical or ellipsoidal conidia that are clustered in chains or acervuli. The hyphae are septate and can form conidia at the tips of the conidiophores, while the colonies exhibit a velvety or cotton-like texture, with variable colors including pink, red, or orange [8,15]. Studies are being conducted on the antifungal activity (*in vitro*) of plant extracts for the control of anthracnose [28].

Plant diseases, such as wilting and root rot, are caused by *Fusarium* spp.; certain species produce mycotoxins that are detrimental to living organisms [29]. They belong to the *Nectriaceae* family, order *Hypocreales*, class *Sordariomycetes*, and phylum *Ascomycota* [30]. The conidia can be white, pink, red, or purple, elongated, and often appear in chains. Macroconidia and microconidia can be observed in the septate hyphae, and the texture can be cottony, velvety, or powdery [31,32].

The genus *Aspergillus* spp. belongs to the phylum *Ascomycota* and class *Eurotiomycetes* and is widely recognized for both its industrial relevance and its implications for health. Microscopically, *Aspergillus* spp. is characterized by conidia arranged in chains or clusters, supported by erect and robust conidiophores. The hyphae are septate, hyaline, or pigmented, and the mycelium is usually dense and well developed. The colonies exhibit a considerable diversity of colors, including green, black, white, and yellow, depending on the species, with their texture varying from smooth and velvety to cottony. These morphological characteristics allow for reliable identification through microscopic analysis and cultures in specific media [33,34].

The review identifies that, although there are previous studies on the antimicrobial properties of EOs, there is a lack of specific research evaluating their efficacy against post-harvest banana fungi (*Musa paradisiaca*), particularly in species such as *Cladosporium* spp., *Colletotrichum* spp., *Lasiodiplodia* spp., and *Fusarium* spp., which is important due to the economic impact these diseases generate in the agricultural sector [35,36]. Therefore, the following research question has been formulated: Can commercially available EOs effectively inhibit the growth of fungi isolated from banana peels under *in vitro* and *ex vivo* conditions? There is a hypothesis that certain EOs, such as cinnamon and clove oils, exhibit significant antifungal activity at specific concentrations, which could provide a sustainable alternative for post-harvest fungal management.

This study addresses a critical gap in post-harvest management by evaluating the antifungal potential of five commercially available essential oils, rosemary, clove, thyme, cinnamon, and basil, against fungal pathogens (*Cladosporium* spp., *Colletotrichum* spp., *Lasiodiplodia* spp., and *Fusarium* spp.) isolated from banana peels (*Musa paradisiaca*). The findings provide valuable information on the efficacy of natural antifungal agents, contributing to the development of sustainable and low-impact agricultural practices. This research not only offers an alternative to chemical fungicides but also supports the global push towards reducing the use of synthetic pesticides in favor of environmentally friendly solutions.

## 2. Materials and Methods

### 2.1. Isolation and Purification of Microorganisms

The isolation and purification of pathogenic fungi was carried out using potato dextrose agar (PDA) (Difco™, Detroit, MI, USA) medium. This medium is widely recognized in microbiology for its ability to promote fungal growth. To suppress bacterial growth, chloramphenicol (Merck, Quito, Ecuador) was added. A stock solution of chloramphenicol at 0.5% (5 mg/mL) was prepared, and the final concentration was adjusted to 0.5 g/L [37].

Approximately 20 g of visibly infected banana peels was taken and washed twice with sterile distilled water to remove superficial contaminants, discarding the water used in each wash. Subsequently, the fragments of the banana peels were placed in an Erlenmeyer flask containing 200 mL of a 0.05% (*v*/*v*) Tween 80 solution (Merck, Ecuador). The mixture was vortexed for 2 min to achieve the initial dilution. From the initial dilution, four serial dilutions were prepared using a 0.1% solution of the previous dilution, ensuring homogeneity through stirring [8].

From each dilution, 0.1 mL was taken and inoculated onto Petri plates containing PDA medium supplemented with 0.05% chloramphenicol. The inoculated plates were incubated at approximately 25 ± 2 °C and a relative humidity of 75 ± 5% with a photo period of 12 h of light and 12 h of darkness and checked every day. Once the visible fungal colonies were formed, they were individually selected using a sterile loop and subcultured onto new PDA plates each week. This process was repeated until pure cultures were obtained, which were used for macroscopic and microscopic morphological analyses.

### 2.2. Morphological Identification

The purified fungal cultures were evaluated weekly using three replicates after the inoculation of the isolated pathogens in PDA medium for morphological identification. The macroscopic characteristics of the colonies were recorded and analyzed in comparison with information obtained from specialized literature, including books and guides on fungal morphology, to determine the genus of each pathogen [38]. During the analysis, criteria such as the shape of the colony, elevation, edges, and surface appearance were considered, allowing for a precise preliminary identification.

The fungus obtained from the culture was examined in triplicate weekly under the microscope using adhesive tape to collect the aerial mycelium and adhere to a microscope slide. The slide was observed under the microscope with 40× and 60× magnification lenses. The evaluation considered the hyphae, the mycelium, the spores, and the structures observed microscopically.

### 2.3. Molecular Identification Through DNA Sequencing

The molecular identification of fungi isolated from banana rot (*Musa paradisiaca*) was carried out through DNA sequencing. For this purpose, genomic DNA was extracted from pure fungal colonies using the commercial Invitrogen kit (Novogene, Sacramento, CA, USA), following the manufacturer’s instructions. The quality and quantity of the extracted DNA were evaluated using spectrophotometry with the Nanodrop spectrophotometer (Thermo Fisher Scientific, Waltham, MA, USA) and 1% agarose gel electrophoresis. A fragment of ribosomal DNA corresponding to the ITS region was amplified (Internal Transcribed Spacer), using universal primers ITS1 (5′-TCCGTAGGTGAACCTGCGG-3′) and ITS4 (5′-TCCTCCGCTTATTGATATGC-3′) [39].

The PCR products were visualized on a 1.5% agarose gel stained with ethidium bromide under UV light. The purified amplicons were sent for sequencing at Macrogen Inc. (Seoul, South Korea). The obtained sequences were analyzed using the software BioEdit version 7.0 [40] and compared with public databases, such as NCBI GenBank (https://www.ncbi.nlm.nih.gov/genbank/, accessed on 4 March 2025), through the algorithm BLASTn, to determine the identity of the fungi. The species were identified based on a similarity of ≥98% compared to deposited sequences.

Phylogenetic analyses were performed by aligning ITS sequences with ClustalW. Phylogenetic trees were generated using both the unweighted pair group method with arithmetic mean (UPGMA) and the neighbor-joining method in MEGA v11 [41]. Pairwise identity matrices and genetic distances were computed based on the Tamura-Nei model. Clade support was assessed through 1000 bootstrap replicates. This methodology is supported by previous studies highlighting the reliability of ITS sequences for fungal identification and phylogeny [10,21,42].

### 2.4. Ex Vivo Fungal Activity

The *ex vivo* analysis was performed with bananas (*Musa paradisiaca*) harvested at physiological maturity, selected for uniformity in size, color, and absence of visible damage. The fruits were disinfected by immersing them in a 1% sodium hypochlorite solution for 5 min, rinsed with sterile distilled water, and dried at room temperature [43].

Inoculum was prepared with a concentration adjusted to 10^6^ conidia/mL for each genus, and the fruits were inoculated using the wound method to evaluate the effect of the antifungal treatments. For the evaluation of fungal growth in 20 banana samples, 100 µL of the adjusted fungal inoculum was deposited to maintain them at approximately 13 ± 1 °C and a relative humidity of 92 ± 3%. Observations were made at regular intervals to record the lesion diameter in millimeters using a millimeter ruler and to record fungal growth by measuring the growth diameter, with four replicates for each treatment [44].

A one-way analysis of variance (ANOVA) was conducted to evaluate significant differences in growth among species, followed by Tukey’s HSD post-hoc test with a significance level of α = 0.05.

### 2.5. In Vitro Antifungal Activity with Essential Oils

The antifungal activity was analyzed under controlled laboratory conditions at approximately 25 ± 2 °C and a relative humidity of 75 ± 5%, with a photo period of 12 h of light and 12 h of darkness, and checked every day. We used EOs from batch 20230516 supplied by Green Harmony, a company specializing in the production and distribution of pure and natural oils.

The oregano EO is sourced from Turkey, cinnamon from Sri Lanka, clove from Indonesia, thyme from Japan, basil from India, and rosemary from Spain [45,46]. These were obtained from specific parts of the plants through steam distillation, a standard process for extracting bioactive compounds.

Oregano oil (*Origanum vulgare*) was obtained from dry leaves, while the cinnamon (*Cinnamomum verum*) was obtained from dried bark and the clove (*Syzygium aromaticum*) was obtained from dried flower buds. In the case of rosemary (*Salvia rosmarinus*), basil (*Ocimum basilicum*), and thyme (*Thymus vulgaris*), the oils were extracted from fresh leaves and flowers [47].

Essential oils (EOs) derived from cinnamon are primarily composed of cinnamaldehyde (60–70%) and eugenol (5–15%), both of which are well documented for their antimicrobial and antioxidant activities.

Basil essential oils are notable for their elevated concentrations of eugenol (50–70%), linalool (10–15%), and methyl chavicol (5–15%), compounds with recognized antimicrobial, anti-inflammatory, and antioxidant functions.

In oregano EOs, carvacrol is the predominant constituent (60–80%), a phenolic compound widely associated with antimicrobial and antioxidant efficacy. Additional components such as thymol (5–10%), p-cymene (5–10%), and γ-terpinene (2–5%) act synergistically to suppress microbial proliferation.

Clove essential oils are characterized by a high eugenol content (70–85%), along with notable amounts of acetyl eugenol (5–15%) and β-caryophyllene (5–12%), which together confer potent antifungal and antibacterial effects.

Thyme oils are rich in thymol (30–50%) and carvacrol (10–20%), supported by p-cymene (15–25%) and γ-terpinene (10–15%), contributing collectively to their strong antifungal potential.

Rosemary essential oils predominantly contain 1,8-cineole (20–50%), carnosol (5–15%), rosmarinic acid (2–5%), and α-pinene (10–20%), all of which are linked to antioxidant, antimicrobial, and potential neuroprotective properties.

Each EO was initially mixed with a 0.05% Tween 80 solution to form a homogeneous emulsion, and then serial dilutions were prepared to achieve concentrations of 200, 400, 600, 800, and 1000 ppm. These solutions were added to the cooled PDA medium before solidifying, following standard laboratory procedures.

The pathogens were inoculated to identify the most effective concentration. Visual inspections were conducted in triplicate, with four subsamples per replicate for each microorganism. Assessments were performed every 24 h to determine the percentage of mycelial growth inhibition and to evaluate the antifungal effectiveness of the EOs applied [10,48].

As a negative control, a PDA medium containing 0.05% Tween 80 but without EOs was used to evaluate the natural growth of fungi without treatment. This control allowed us to evaluate the efficacy of the EOs by comparing the growth inhibition observed in the treated samples with the untreated controls.

The *in vitro* antifungal activity of six EOs (cinnamon, clove, basil, oregano, rosemary, and thyme) was evaluated against five genera of phytopathogenic fungi: *Cladosporium* spp., *Lasiodiplodia* spp., *Colletotrichum* spp., *Fusarium* spp., and *Aspergillus* spp. The assay was performed using the solid medium dilution method with PDA supplemented with the essential oils at concentrations of 200, 400, 600, 800, and 1000 ppm. Inhibition was recorded as positive (−) when no mycelial growth was observed and negative (+) when growth occurred.

## 3. Results

### 3.1. Morphological Identification

**Figure 1:**
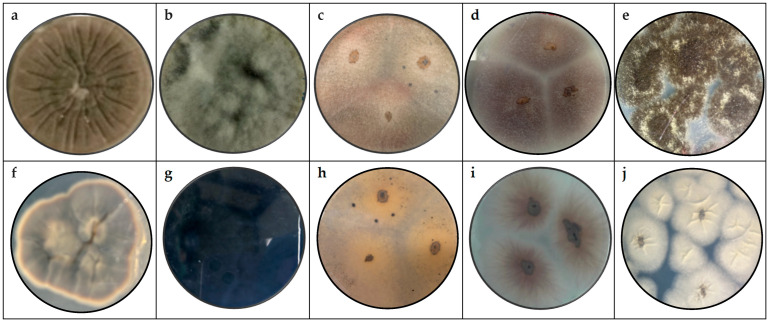
Macroscopy images of (**a**) *Cladosporium* spp., (**b**) *Lasiodiplodia* spp., (**c**) *Colletotrichum* spp., (**d**) *Fusarium* spp., and (**e**) *Aspergillus* spp., considering the appearance of the front side; and (**f**) *Cladosporium* spp., (**g**) *Lasiodiplodia* spp., (**h**) *Colletotrichum* spp., (**i**) *Fusarium* spp., and (**j**) *Aspergillus* spp. considering the appearance of the reverse side.

Figure 1 shows the pure fungi isolated from banana peel rot on a selective medium (PDA + chloramphenicol) and stored in PDA at approximately 25 °C, with macroscopic images of the front and reverse sides of *Cladosporium* spp., *Lasiodiplodia* spp., *Colletotrichum* spp., *Fusarium* spp., and *Aspergillus* spp.

**Figure 2:**
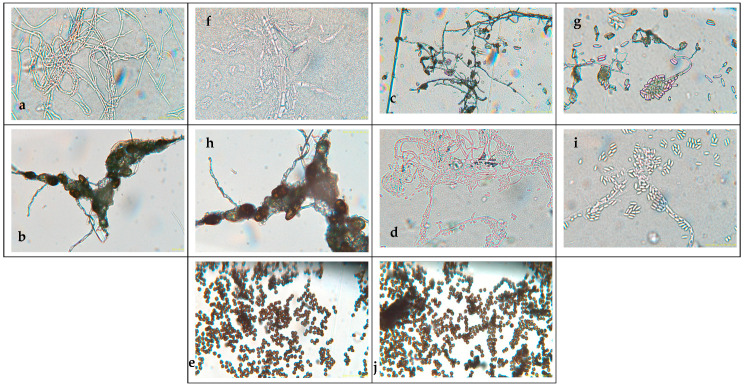
Microscopy images of (**a**) *Cladosporium* spp., (**b**) *Lasiodiplodia* spp., (**c**) *Colletotrichum* spp., (**d**) *Fusarium* spp., and (**e**) *Aspergillus* spp. at 40×; and (**f**) *Cladosporium* spp., (**g**) *Lasiodiplodia* spp., (**h**) *Colletotrichum* spp., (**i**) *Fusarium* spp., and (**j**) *Aspergillus* spp. at 60×.

Figure 2 presents the aerial mycelium of the fungi. These observations provide visual information that complements the morphological analysis and microscopic images observed under 40× and 60× magnifications.

### 3.2. Molecular Identification Through DNA Sequencing

**Figure 3:**
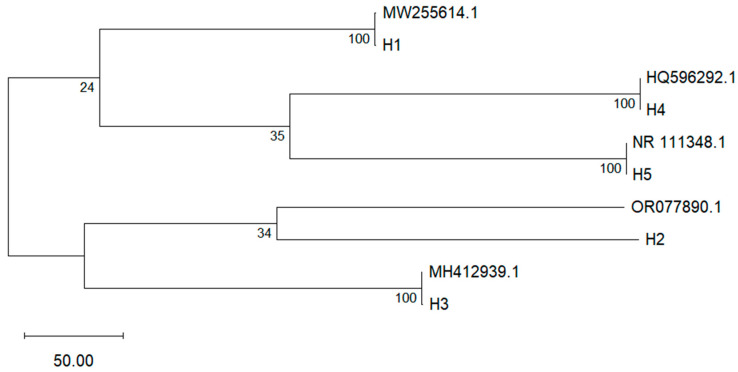
Phylogenetic tree based on ITS region sequences of fungal isolates associated with *Musa paradisiaca*.

The phylogenetic relationships among the fungal isolates were inferred from ITS region sequences using the neighbor-joining method (Figure 3). Five distinct fungus types (H1–H5) were identified, with strong bootstrap support values at nodes. *Cladosporium cladosporioides* (MW255614.1, H1) was clearly separated from the remaining isolates, forming a distinct clade. Meanwhile, *Colletotrichum musae* (HQ596292.1, H4) and a fungal sequence (NR_111348.1, H5) grouped together with 100% support. Similarly, *Fusarium ophioides* (MH412939.1, H3) and *Lasiodiplodia theobromae* (OR077890.1, H2) clustered in different clades. These results suggest significant genetic divergence among the isolates, consistent with their classification into different genera.

Table 1 summarizes the results obtained from sequencing the ITS region of fungi isolated from banana peels. It includes the names of the identified organisms, the genetic fragments used in sequencing, and the percentage obtained through a comparison with the database. The high similarity of the sequences (≥98%) confirms the accuracy of the identification of the fungal genera and species present in the analyzed samples [43,49].

A pairwise distance matrix based on ITS sequences was constructed to assess genetic divergence among the fungal isolates (Table 2). The lowest genetic distance (0.000) was observed between *OR077890.1* (H2) and both *MH412939.1* (H3) and *HQ596292.1* (H4), indicating high sequence similarity. In contrast, the highest divergence was found between *MW255614.1* (H1) and *NR_111348.1* (H5), with 7.225–7.234.

### 3.3. Fungal Activity Ex Vivo

**Figure 4:**
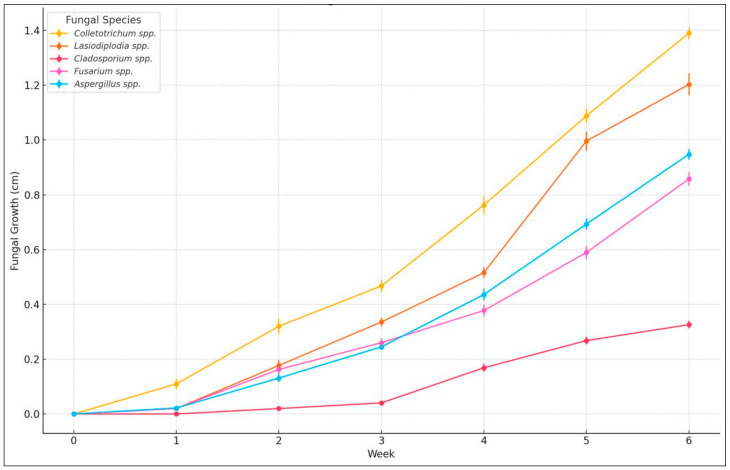
Fungal growth (cm) over 6 weeks in 20 banana samples inoculated with *Cladosporium* spp., *Lasiodiplodia* spp., *Colletotrichum* spp., *Fusarium* spp., and *Aspergillus* spp., stored at approximately 13 °C and a relative humidity of 95%.

The severity of the fungal infection was assessed through *ex vivo* analysis. Figure 4 shows the evaluation of fungal growth in banana samples, which were monitored over a period of 6 weeks. The analysis focused on identifying and evaluating the growth of *Cladosporium* spp., *Lasiodiplodia* spp., *Colletotrichum* spp., *Fusarium* spp., and *Aspergillus* spp.

A one-way analysis of variance (ANOVA) was conducted to assess the effect of treatments and fungal species on *ex vivo* growth in *Musa paradisiaca* samples during post-harvest storage. The results revealed statistically significant differences among treatments (*p* < 0.05), indicating that both the type of treatment and its concentration had a significant influence on fungal development. Furthermore, the ANOVA showed highly significant differences among the fungal species evaluated (F = 34.77, *p* < 0.001).

Tukey’s HSD post hoc test (α = 0.05) revealed that *Colletotrichum* spp. exhibited the highest growth, differing significantly from all other species except *Lasiodiplodia* spp. In contrast, *Cladosporium* spp. displayed the lowest growth, with values significantly lower than those of all other species (*p* < 0.05). No significant differences were observed between *Aspergillus* spp. and *Fusarium* spp. (*p* > 0.99). Assumptions of normality and homogeneity of variances were verified based on data symmetry and the similarity of observed standard deviations.

### 3.4. In Vitro Antifungal Activity with Essential Oils

**Figure 5:**
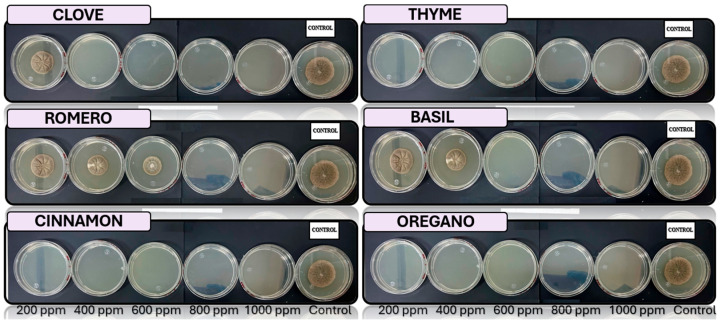
*In vitro* analysis of *Cladosporium* spp. in PDA medium supplied with basil, cinnamon, clove, oregano, rosemary, and thyme essential oils at various concentrations of 200, 400, 600, 800, and 1000 ppm (*n* = 12).

**Figure 6:**
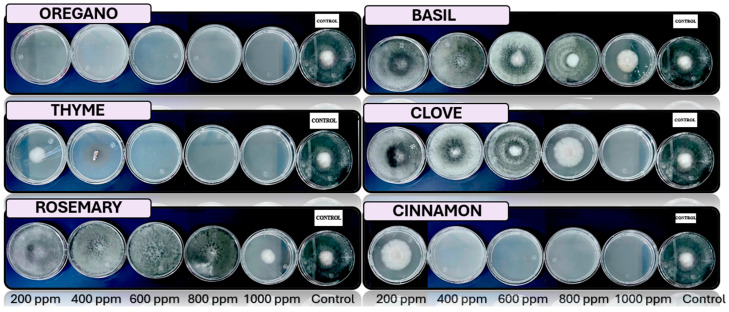
*In vitro* analysis of *Lasiodiplodia* spp. in PDA medium supplied with basil, cinnamon, clove, oregano, rosemary, and thyme essential oils at various concentrations of 200, 400, 600, 800, and 1000 ppm (*n* = 12).

**Figure 7:**
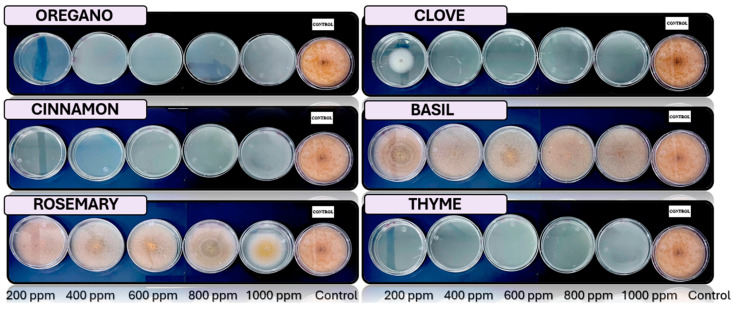
*In vitro* analysis of *Colletotrichum* spp. in PDA medium supplied with basil, cinnamon, clove, oregano, rosemary, and thyme essential oils at various concentrations of 200, 400, 600, 800, and 1000 ppm (*n* = 12).

**Figure 8:**
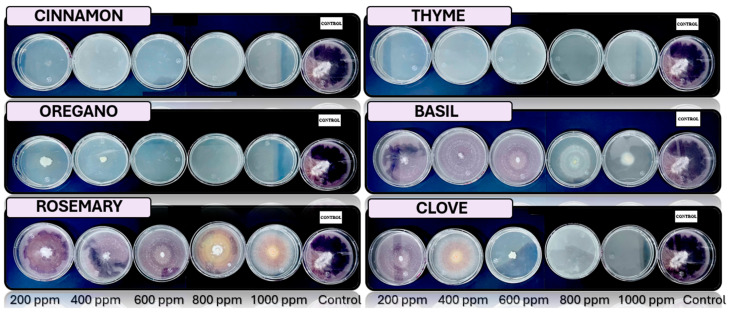
*In vitro* analysis of *Fusarium* spp. in PDA medium supplied with basil, cinnamon, clove, oregano, rosemary, and thyme essential oils at various concentrations of 200, 400, 600, 800, and 1000 ppm (*n* = 12).

**Figure 9:**
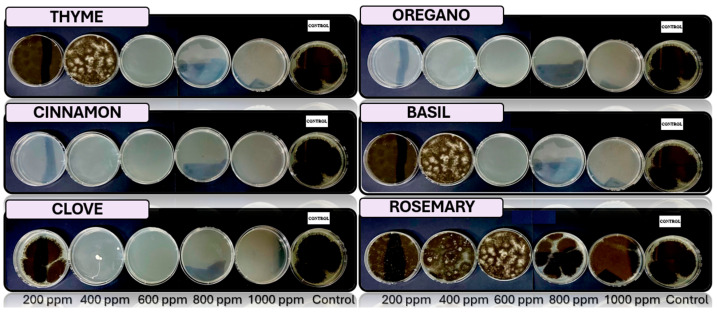
*In vitro* analysis of *Aspergillus* spp. in PDA medium supplied with basil, cinnamon, clove, oregano, rosemary, and thyme essential oils at various concentrations of 200, 400, 600, 800, and 1000 ppm (*n* = 4).

Figure 5, Figure 6, Figure 7, Figure 8 and Figure 9 illustrate the growth of *Cladosporium* spp., *Lasiodiplodia* spp., *Colletotrichum* spp., *Fusarium* spp., and *Aspergillus* spp. in PDA medium with EO concentrations of 200, 400, 600, 800, and 1000 ppm. The EOs evaluated include oregano, basil, cinnamon, rosemary, thyme, and clove.

In this study, the antifungal activity of EOs was evaluated *in vitro* against various fungal species. The “+” symbol indicates fungal growth, while the “−” symbol denotes the inhibition of fungal growth at the corresponding concentration.

Table 3 presents the *ex vivo* analysis of antifungal activity, demonstrating that a concentration of 400 ppm of cinnamon EOs effectively inhibits the growth of the five fungal species tested.

**Figure 10:**
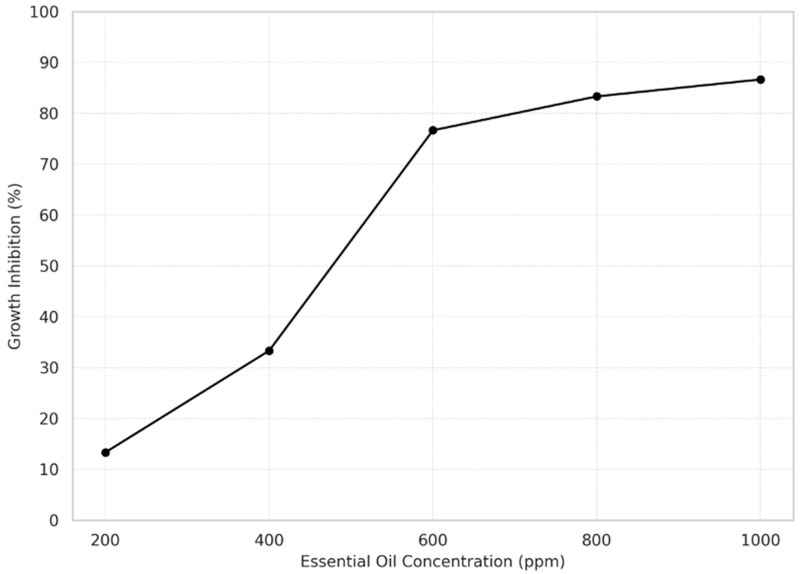
Fungal growth inhibition according to essential oils’ concentration.

A concentration-dependent inhibition was observed across all tested essential oils (Figure 10). Starting at 600 ppm, most oils exhibited over 75% inhibition, reaching a maximum of 96.7% at 1000 ppm.

Cinnamon, oregano, and thyme oils were the most effective, each achieving 23 out of 25 possible inhibitions. In contrast, basil oil showed the lowest antifungal activity with only 16 inhibitions. Among fungal species, *Cladosporium* spp. was the most susceptible (86.7% inhibition), whereas *Aspergillus* spp. was the most resistant (70.0%).

## 4. Discussion

### 4.1. Morphological Identification

Five samples from 14 fungal colonies isolated from infected banana peel were analyzed. Significant differences were observed between the fungal genera in terms of macroscopic morphology and microscopic characteristics.

For *Cladosporium cladosporoides*, the macroscopic analysis shows that it has a powdery appearance, is green on both the front and back, has a velvety, cottony, and crater-like (crateriform) appearance [50], and has colonies with a velvety texture and olive-green color. Microscopically, it has conidia in chains and septate hyphae [47].

*Lasiodiplodia* spp.’s macroscopic characteristics include a woolly appearance with a black to gray color, rapid growth, and a rough surface. These characteristics are similar to those reported when evaluating banana crown rot [51]. *Lasiodiplodia brasiliensis* and * Lasiodiplodia theobromae*‘s macroscopic characteristics include colonies that are dark brown in color, containing ellipsoidal conidia and thick walls, and are known for their association with fruit rot [35].

*Colletotrichum* spp. exhibits pigmented mycelia with a beige-orange color, characterized by a slightly raised, flat elevation, and is identified as the most significant fungal agent affecting bananas [52]. *Colletotrichum musae* has orange and smooth colonies, with conidia in the form of oval and elongated shapes arranged in short chains along the conidiophore, with septate and branched hyphae [53]. This species is the main causal agent of anthracnose in bananas, as noted by other authors [36,54].

The post-harvest deterioration of bananas is due to the presence of *Fusarium* spp. of various species [31,55]. This species exhibits a white and purple coloration with irregular edges and a cottony appearance. *Fusarium ophioides* has white to purple colonies and a sickle shape, consistent with other studies [9].

*Aspergillus niger* has dense black colonies, with conidia in clusters, characteristics similar to those reported in studies on post-harvest pathogens in tropical fruits [43]. *Aspergillus* spp. has various applications in biotechnology, including the secretion of organic acids, proteins, enzymes, and secondary metabolites due to its prolific nature. The genus is suspected based on its appearance on PDA, on which it appears as round black spores with a rough texture, with black coloration on the front and white on the back [56].

### 4.2. Molecular Identification Through DNA Sequencing

Molecular identification through the sequencing of the ITS region of ribosomal DNA enabled the confirmation of the presence of the five fungal genera isolated (H1, H2, H3, H4, and H5) from banana peel rot. The ITS region has proven to be a reliable genetic marker for identifying fungi. This study enabled the identification of species with similar morphological characteristics, such as *Lasiodiplodia brasiliensis* and *L. theobromae*, which would not be possible through macroscopic or microscopic analysis alone [57]. Previous research has highlighted that the ITS region offers a high sensitivity for identifying pathogens associated with tropical crops, supporting its application [35,57]. This approach complemented the morphological macroscopic and microscopic observations, providing a robust and precise tool to differentiate species with similar characteristics.

The phylogenetic analysis based on ITS sequences revealed taxonomic distinctions among the fungal isolates associated with *Musa paradisiaca*. The separation of *Cladosporium cladosporioides* from the other taxa reflects its genetic distance and ecological specificity. The close clustering of *Colletotrichum musae* and the fungal isolate suggests an ITS region, potentially indicating phylogenetic proximity or shared ecological adaptations. The clade formed by *Fusarium ophioides* and *Lasiodiplodia theobromae* aligns with that of the ITS markers, resolving relationships among phytopathogenic fungi.

These findings reinforce the value of ITS-based phylogeny as a tool for elucidating evolutionary relationships and validating morphological identifications in post-harvest disease research. The genetic variability observed among haplotypes also provides insight into the potential diversity present in banana cultivation systems.

The ITS sequencing showed a 99.62% similarity with *Cladosporium cladosporoides*, confirming identicality. This fungus is widely known as an opportunistic colonizer of post-harvest fruits. Under high-humidity conditions, it is reported as an agent that contributes to the superficial deterioration of fruits, and its impact is less aggressive compared to other genera such as *Colletotrichum* spp. [23]. Studies conducted on tropical fruits demonstrated that *C. cladosporoides* persists on the surface of the fruit without causing significant damage, but under stress conditions, it can act as a secondary pathogen [23].

The molecular identification of *Lasiodiplodia theobromae* with a similarity of 100% in ITS confirms its association with post-harvest diseases in bananas. These species are known for causing rot in tropical crops [58]. *L. theobromae* is identified as one of the main causes of crown rot in bananas, with significant economic implications [59]. Studies conducted in mango plantations in Brazil highlight their ability to produce toxic secondary metabolites that exacerbate the severity of infections [59].

The 99.56% similarity obtained for *Colletotrichum musae* confirmed its role as the main causal agent of banana anthracnose. This pathogen is capable of colonizing both superficial and deep tissues, leading to the formation of dark spots that compromise the fruit’s quality during storage [53]. In Ecuador, recent studies document that *C. musae* accounts for up to 50% of post-harvest losses in exported bananas, especially under high-humidity conditions [4]. The pathogenicity of this fungus is associated with the production of cell-wall-degrading enzymes, such as cellulases and pectinases, which allow it to quickly invade the fruit’s tissues [15].

For the identification of *Fusarium ophioides* (a 99.78% similarity), this species has been less studied in the context of post-harvest banana diseases. *Fusarium* spp. is mainly known for causing Panama disease in bananas (*Fusarium oxysporum f.* sp. *cubense*). *F. ophioides* has been identified as a frequent contaminant in fruits stored under humid conditions [60]. Its ability to produce mycotoxins, such as fumonisins, could pose a risk both to fruit quality and human health [61].

The similarity of 99.83% with *Aspergillus niger* confirmed its identification as a frequent contaminant in stored bananas. This fungus is recognized for its ability to produce mycotoxins and cause black rot in fruits. In high-humidity conditions, *A. niger* can proliferate rapidly, affecting the appearance and quality of fruits [62]. In a study conducted in the Philippines, it was documented that *A. niger* was responsible for 20% of the losses in bananas stored for more than 10 days at temperatures above 20 °C [63].

The pairwise distance matrix confirms the clustering patterns observed in the phylogenetic tree, with minimal divergence among isolates H2, H3, and H4, suggesting a close evolutionary relationship or possible intraspecific variation. The strong similarity among these isolates may reflect conserved ITS regions or shared ecological niches within post-harvest banana pathogens.

### 4.3. Ex Vivo Fungal Activity

After the characterization of the isolated fungi, the growth patterns and severity were determined. The inhibition index was calculated for 20 samples by measuring the fungal growth diameter on inoculated bananas. In Figure 4, the analysis of the average values with their respective deviations is presented. The *p*-value of the F-test is less than 0.05, indicating a statistically significant difference between the means of the five variables at a 95.0% confidence level. It is concluded that the pathogens were evaluated in the following order of severity, from highest to lowest: *Colletotrichum* spp., *Lasiodiplodia* spp., *Aspergillus* spp., *Fusarium* spp., and *Cladosporium* spp.

In this analysis, *Colletotrichum* spp. presented the greatest impact, followed by *Lasiodiplodia* spp. and * Fusarium* spp., while *Aspergillus* spp. and * Cladosporium* spp. had a lesser effect. The severity classification is consistent with previous studies that highlight *Colletotrichum* spp. as the most aggressive pathogen in tropical fruits due to its rapid colonization and ability to produce hydrolytic enzymes [36,47].

The differences observed between genders could be attributed to their ability to adapt to post-harvest conditions. For example, it was reported that *Lasiodiplodia* spp. is known for its tolerance to high temperatures and its ability to colonize weakened tissues [35]. *Fusarium* spp. tends to cause more severe damage in high-humidity conditions [54].

The analysis of variance (ANOVA) revealed statistically significant differences in fungal growth among the evaluated species (F = 34.77, *p* < 0.001). Tukey’s HSD post-hoc test showed that *Colletotrichum* spp. exhibited the highest growth and differed significantly from all other species except *Lasiodiplodia* spp. *Cladosporium* spp. displayed the lowest growth, which was significantly lower than that of all other species (*p* < 0.05). No significant difference was observed between *Aspergillus* spp. and *Fusarium* spp. (*p* > 0.99).

### 4.4. In Vitro Antifungal Activity with Essential Oils

The fungi were evaluated (*Cladosporium* spp., *Lasiodiplodia* spp., *Colletotrichum* spp., *Fusarium* spp., and *Aspergillus* spp.) for the effectiveness of EOs considering growth, measuring the fungal growth diameter in PDA medium under controlled conditions [64]. The most effective concentration was identified through the analysis conducted in triplicate with four replicates, and the Petri dishes were examined every day to determine the inhibition percentage and the effectiveness of the EOs at the established concentration [65].

The findings demonstrate the potential of essential oils as natural antifungal agents. The high efficacy observed for cinnamon, oregano, and thyme oils aligns with previous studies reporting their richness in phenolic compounds such as cinnamaldehyde, carvacrol, and thymol, which are known to disrupt fungal membrane integrity [10,21]. The differential susceptibility among fungal species may be attributed to structural differences in their cell walls and enzymatic defense mechanisms. These results underscore the importance of selecting specific oils based on the target pathogen.

The antifungal efficacy of EOs was further explored through logistic regression analysis, which confirmed that both the type of essential oil and its concentration were significantly associated with the probability of fungal inhibition (*p* < 0.001). The model demonstrated excellent goodness of fit, as indicated by a Pseudo R^2^ value of 0.77, reflecting a strong predictive relationship between treatment factors and inhibition outcomes.

The inhibition data were processed to calculate the percentage of fungal growth inhibition at each concentration level. These values were subsequently visualized through a concentration–response curve (Figure 10), generated using Statgraphics Centurion software, XVI, 16.1.03. The graph presents the inhibition percentage on the *Y*-axis and the essential oil concentration (ppm) on the *X*-axis, clearly illustrating the positive association between increasing concentrations and enhanced inhibitory effects. These results reinforce the dose-dependent antifungal activity of the evaluated essential oils and highlight the importance of both qualitative and quantitative optimization in post-harvest fungal control strategies.

Oregano contains bioactive compounds such as carvacrol, thymol, and phenolic acids, which exhibit antimicrobial and antioxidant properties. Cinnamon contains cinnamaldehyde, eugenol, and flavonoids, which have potential anti-inflammatory, antioxidant, and glucose-regulating effects. Clove contains eugenol, acetyl eugenol, and flavonoids, which act as analgesic, antimicrobial, and antioxidant agents [12,14]. Cinnamon EO was used as a bioactive compound to extend the shelf life of strawberries as a post-harvest treatment [66].

Basil contains bioactive compounds such as eugenol, rosmarinic acid, and flavonoids, which exhibit antioxidant and anti-inflammatory properties. Thyme contains thymol, carvacrol, and flavonoids, which generate antibacterial and antioxidant properties, while also improving digestion. Rosemary contains rosmarinic acid, carnosol, and flavonoids, which have antioxidant, anti-inflammatory, and potential neuroprotective effects [13,15,16].

In this study, the antifungal activity of commercially acquired EOs (EO) of oregano (*Origanum vulgare*), rosemary (*Salvia rosmarinus*), clove (*Syzygium aromaticum*), thyme (*Thymus vulgaris*), cinnamon (*Cinnamomum verum*), and basil (*Ocimum basilicum*) was determined.

Cinnamon EO was the most effective in this study, achieving inhibition rates of over 90% against all fungi at 400 ppm. Its efficacy is mainly attributed to cinnamaldehyde, a compound with strong antimicrobial activity. In a study, authors highlighted that it effectively inhibits *Colletotrichum musae*, responsible for anthracnose in bananas because cinnamaldehyde destabilizes the fungal cell membranes, causing the loss of essential intracellular components [67]. Other studies have reported that it is effective against a wide range of fungi, including *Fusarium* spp. and *Aspergillus* spp., due to its ability to alter the permeability of the cell membrane [10].

Clove EO showed moderate activity, inhibiting between 70% and 80% of fungal growth. Its main active compound, eugenol, is known for its antimicrobial and antifungal properties. Studies have shown that it is as effective as synthetic fungicides such as thiabendazole against *Colletotrichum* spp. in bananas [11]. Other studies have confirmed that the eugenol present in clove EOs inhibits the development of post-harvest pathogens, especially *Lasiodiplodia theobromae* [67].

Thyme EO exhibited significant efficacy, especially against *Fusarium* spp. and *Aspergillus* spp., with inhibitions of up to 75%. Its activity is due to compounds such as thymol and carvacrol, which alter the cellular permeability of the fungi. Research has reported that it inhibits the sporulation of *Fusarium* spp. and significantly reduces the development of *Aspergillus niger* on tropical fruits [9,68]. It was found that thymol and carvacrol disrupt fungal cell membranes, causing osmotic imbalances [69].

Oregano EO inhibited between 60% and 75% of fungal growth, showing notable activity against *Lasiodiplodia* spp. Its main components, carvacrol and thymol, act synergistically to enhance its antifungal efficacy. In analyses, it was documented that it significantly reduces the development of *Lasiodiplodia theobromae* in stored bananas, extending their shelf life [70]. It was also noted that oregano EO is effective against *Fusarium* spp. due to its ability to interact with the lipid membranes of the fungi [10].

Rosemary EO showed more moderate activity, with inhibitions ranging from 50% to 65%, depending on the fungal genus. This effect is attributed to compounds such as 1,8-cineole and camphor. Research has shown that it has a fungistatic effect against pathogens such as *Colletotrichum* spp., but its efficacy depends on the concentration used [43]. It was suggested that 1,8-cineole present in rosemary EO could inhibit fungal development by altering cellular respiration [69].

Basil EO exhibited more limited antifungal activity, with inhibitions ranging from 40% to 60%. Although it contains bioactive compounds such as linalool and estragole, its efficacy depends on the concentration used and the fungal genus. Other studies have reported that it partially inhibits the development of *Aspergillus niger* and *Fusarium* spp. in stored bananas [71]. The variability in the chemical composition of basil EO may influence its antifungal effectiveness [10].

The results of this study support the use of EOs as sustainable alternatives for post-harvest management in bananas. Each EO exhibits variable efficacy, depending on its chemical composition and the target pathogen. These observations are consistent with previous research, highlighting the importance of selecting an appropriate EO and optimizing its application conditions.

## 5. Conclusions

This study highlighted the antifungal potential of EOs from oregano (*Origanum vulgare*), rosemary (*Salvia rosmarinus*), clove (*Syzygium aromaticum*), thyme (*Thymus vulgaris*), cinnamon (*Cinnamomum verum*), and basil (*Ocimum basilicum*) against fungi isolated from banana peels (*Musa paradisiaca*). Cinnamon EO was the most effective, significantly inhibiting fungal growth at a concentration of 400 ppm, both *in vitro* and *ex vivo*. The fungal strains were classified according to their severity, with *Colletotrichum musae* being the most aggressive, followed by *Lasiodiplodia theobromae*, *Aspergillus niger*, *Fusarium ophioides*, and *Cladosporium cladosporoides*. These results support the use of EOs as sustainable alternatives to synthetic fungicides in the post-harvest management of bananas.

In the *in vitro* study to evaluate the efficacy of EOs, cinnamon was effective at 400 ppm, thyme at 600 ppm, and clove at 1000 ppm, while basil and rosemary did not inhibit the growth of the analyzed pathogens. The control of *Colletotrichum musae* was achieved with 200 ppm of oregano and thyme, 400 ppm of cinnamon, and 1000 ppm of clove. *Lasiodiplodia theobromae* was controlled with 200 ppm of cinnamon and oregano, 600 ppm of thyme, and 1000 ppm of clove. *Aspergillus niger* was controlled with 200 ppm of cinnamon and oregano and with 600 ppm of clove, basil, and thyme. *Fusarium ophioides* was controlled with 200 ppm of cinnamon and thyme and with 800 ppm of clove and oregano. *Cladosporium cladosporoides* was controlled with 200 ppm of oregano and thyme, 400 ppm of cinnamon and clove, 600 ppm of basil, and 800 ppm of rosemary.

Future research should focus on evaluating Eos’ economic viability, large-scale application, and potential impacts on fruit quality, maximizing their potential in sustainable agricultural practices.

## Figures and Tables

**Table 1:** Molecular identification of fungal isolates from *Musa paradisiaca* based on ITS sequences and NCBI database comparison.

Organism	Fragment	NCBI	% Identity
*Cladosporium cladosporoides*	ITS	MW255614.1	99.62%
*Lasiodiplodia theobromae*	ITS	OR077890.1	100.00%
*Colletotrichum musae*	ITS	HQ596292.1	99.56%
*Fusarium ophioides*	ITS	MH412939.1	99.78%
*Aspergillus niger*	ITS	NR_111348.1	99.83%

**Table 2:** Sequencing results for fungi isolated from the samples of *Musa paradisiaca*.

	MW255614.1	H1	OR077890.1	H2	MH412939.1	H3	HQ596292.1	H4	NR_111348.1	H5
MW255614.1	ID	0.003	6.248	6.248	6.248	6.248	6.248	6.248	7.225	7.225
H1	0.005	ID	6.256	6.256	6.256	6.255	6.256	6.256	7.234	7.234
OR077890.1	0.378	0.380	ID	0.000	0.000	0.002	0.000	0.000	6.398	6.398
H2	0.378	0.380	0.000	ID	0.000	0.002	0.000	0.000	6.398	6.398
MH412939.1	0.378	0.380	0.000	0.000	ID	0.002	0.000	0.000	6.398	6.398
H3	0.380	0.382	0.002	0.002	0.002	ID	0.002	0.002	6.397	6.397
HQ596292.1	0.378	0.380	0.000	0.000	0.000	0.002	ID	0.000	6.398	6.398
H4	0.378	0.380	0.000	0.000	0.000	0.002	0.000	ID	6.398	6.398
NR_111348.1	0.460	0.462	0.417	0.417	0.417	0.421	0.417	0.417	ID	0.000
H5	0.460	0.462	0.417	0.417	0.417	0.421	0.417	0.417	0.000	ID

**Table 3:** Evaluation of *in vitro* antifungal activity of essential oil against *Cladosporium* spp., *Lasiodiplodia* spp., *Colletotrichum* spp., *Fusarium* spp., and *Aspergillus* spp. using oregano, rosemary, clove, thyme, cinnamon, and basil essential oils.

Essential Oil	Fungus	Concentration [ppm]				
		200	400	600	800	1000
Cinnamon	*Cladosporium* spp.	+	−	−	−	−
	*Lasiodiplodia* spp.	−	−	−	−	−
	*Colletotrichum* spp.	−	−	−	−	−
	*Fusarium* spp.	−	−	−	−	−
	*Aspergillus* spp.	−	−	−	−	−
Clove	*Cladosporium* spp.	+	−	−	−	−
	*Lasiodiplodia* spp.	+	+	+	+	−
	*Colletotrichum* spp.	+	−	−	−	−
	*Fusarium* spp.	+	+	+	−	−
	*Aspergillus* spp.	+	+	−	−	−
Basil	*Cladosporium* spp.	+	+	−	−	−
	*Lasiodiplodia* spp.	+	+	+	+	+
	*Colletotrichum* spp.	+	+	+	+	+
	*Fusarium* spp.	+	+	+	+	+
	*Aspergillus* spp.	+	+	−	−	−
Oregano	*Cladosporium* spp.	−	−	−	−	−
	*Lasiodiplodia* spp.	−	−	−	−	−
	*Colletotrichum* spp.	−	−	−	−	−
	*Fusarium* spp.	+	+	+	−	−
	*Aspergillus* spp.	−	−	−	−	−
Rosemary	*Cladosporium* spp.	+	+	+	−	−
	*Lasiodiplodia* spp.	+	+	+	+	+
	*Colletotrichum* spp.	+	+	+	+	+
	*Fusarium* spp.	+	+	+	+	+
	*Aspergillus* spp.	+	+	+	+	+
Thyme	*Cladosporium* spp.	−	−	−	−	−
	*Lasiodiplodia* spp.	+	+	−	−	−
	*Colletotrichum* spp.	−	−	−	−	−
	*Fusarium* spp.	−	−	−	−	−
	*Aspergillus* spp.	+	+	−	−	−

## Data Availability

The original contributions presented in this study are included in the article. Further inquiries can be directed to the corresponding author.
